# Poststimulation inhibition of the micturition reflex induced by tibial nerve stimulation in rats

**DOI:** 10.1002/phy2.205

**Published:** 2014-01-28

**Authors:** Yosuke Matsuta, James R. Roppolo, William C. de Groat, Changfeng Tai

**Affiliations:** 1 Department of Urology, University of Pittsburgh, Pittsburgh, Pennsylvania; 2 Department of Pharmacology and Chemical Biology, University of Pittsburgh, Pittsburgh, Pennsylvania

**Keywords:** Neuromodulation, rats, tibial nerve, urinary bladder

## Abstract

The purpose of this study was to determine the effect of tibial nerve stimulation (TNS) on the micturition reflex. Experiments were conducted in 24 rats under urethane anesthesia. A catheter was inserted into the bladder via the bladder dome for saline infusion. A cuff electrode was placed around right tibial nerve for stimulation. TNS (5 Hz, 0.2 msec pulse width) at 2–4 times the threshold (T) intensity for inducing a toe movement was applied either during slow (0.08 mL/min) infusion of the bladder or for 30 min with an empty bladder. TNS had no effect on the micturition reflex when it was applied during slow bladder infusion. However, the 30‐min TNS applied with an empty bladder induced poststimulation inhibition and significantly (*P* < 0.05) increased the bladder capacity to about 140% of prestimulation level in a 50‐min period following the termination of stimulation. The bladder compliance was also significantly (*P* < 0.05) increased after the 30‐min TNS. These results suggest that different mechanisms might exist in acute‐ and post‐TNS inhibition of micturition reflex. The animal model developed in this study will be very useful for further investigations of the neurotransmitter mechanisms underlying tibial neuromodulation of bladder function.

## Abstract (precis)

This study suggests that different mechanisms might exist in acute‐ and poststimulation tibial inhibition of the micturition reflex. The animal model developed in this study is very useful for further investigations of the neurotransmitter mechanisms underlying tibial neuromodulation of bladder function.

## Introduction

Overactive bladder (OAB) is a syndrome characterized as urinary urgency with or without urge incontinence, usually associated with urinary frequency and nocturia (Abrams et al. 2002). OAB has an overall prevalence of 16.5% in the United States with a significant impact on the quality of life (Coyne et al. 2004). Antimuscarinic drugs are the first‐line pharmacotherapy for OAB patients. Although these drugs can successfully reduce OAB symptoms, the drug discontinuance rate is very high due to the adverse effects, cost, and insufficient efficacy (Andersson 2004).

Neuromodulation therapies utilize electrical stimulation to activate specific nerves that can modulate bladder activity and treat OAB. Percutaneous tibial neuromodulation is one of the Food and Drug Administration (FDA)‐approved neuromodulation therapies for OAB, which stimulates the tibial nerve via a fine needle electrode acutely inserted at the ankle above the medial malleolus. Tibial neuromodulation therapy requires 30‐min stimulation per week for an initial 12 weeks followed by stimulation once per month to maintain efficacy. Recent clinical trials have shown that the efficacy of tibial neuromodulation therapy is the same as that of antimuscarinic drugs but with less adverse effect (Peters et al. 2009, 2013). However, the mechanisms underlying tibial neuromodulation therapy are not fully understood.

Based on clinical results, tibial neuromodulation must produce poststimulation inhibition lasting for days or weeks after repeated 30‐min periods of stimulation. An animal model that can mimic the poststimulation inhibition of bladder activity induced by tibial nerve stimulation (TNS) will be very useful for basic science studies aimed at revealing the mechanisms of tibial neuromodulation. Since most studies about the mechanism of action will require invasive or harmful procedures, they are very difficult to perform in human subjects. Therefore, the purpose of this study is to determine if TNS can induce a long‐lasting poststimulation inhibition of bladder activity in a rodent model. Understanding the mechanisms of neuromodulation may provide new targets to develop pharmacotherapies or to improve current neuromodulation therapies for OAB (Andersson 2004).

## Material and Methods

The Animal Care and Use Committee at the University of Pittsburgh approved all protocols involving the use of animals in this study.

### Experimental setup

Experiments were conducted in a total of 24 female Sprague‐Dawley rats weighing 198–280 g (Hilltop Lab Animals, Inc., Scottdale, PA). The animals were anesthetized with isoflurane (2–5% in oxygen) during surgery and then maintained with urethane (1.2 g/kg, subcutaneously) after surgery.

The bladder was exposed via a midline incision in the abdomen. A polyethylene catheter (PE50; Becton Dickinson, Sparks, MD) was inserted into the bladder from the dome with the other end of the catheter connected via a three‐way stopcock to a pump (Harvard Apparatus, Holliston, PA) for saline infusion and a pressure transducer (World Precision Instruments, Sarasota, FL) to record bladder pressure. The tibial nerve was exposed on the medial side of right hindlimb above the ankle. A tripolar cuff electrode (NC223pt; MicroProbe, Inc., Gaithersburg, MD) was applied around the nerve and connected to a stimulator (S48; Grass Technologies, Astro‐Med Inc., West Warwick, RI) via a stimulus isolation unit (PSIU6 Photoelectric Isolation Unit; Grass Technologies, Astro‐Med Inc., West Warwick, RI). The skin was closed at the end of the surgery.

### Stimulation protocol

Uniphasic rectangular pulses (5 Hz frequency, 0.2 msec pulse width) were delivered via the cuff electrode to the tibial nerve. Threshold (T) stimulation intensity was defined as the minimal intensity to induce a toe twitch. Previous studies in rats and cats indicated that TNS intensity greater than 2T is required to inhibit reflex bladder contractions (Tai et al. 2011a; Su et al. 2012) Therefore, we chose to use intensities of 2–4T to inhibit the bladder in this study.

Initially, a cystometrogram (CMG) was performed in all 24 rats by continuously infusing (0.08 mL/min) room temperature saline until intercontraction intervals stabilized. Then, to evaluate the reproducibility three to five control CMGs were performed by stopping the infusion at the initiation of the first micturition contraction. Once the control bladder capacity was determined, a CMG was performed during continuous TNS. Following this TNS CMG, the rats were divided into two groups. In the first group (*N* = 12 rats), TNS was applied for 30 min with the bladder empty. In the second group (*N* = 12 rats), the bladder remained empty for 30 min without applying TNS. After the 30 min, five control CMGs without TNS were performed during a 1‐h period in both groups to evaluate any change in bladder capacity. The bladder was emptied at the end of each CMG and a 5‐min waiting period was inserted between CMGs.

### Data analysis

The measured CMG parameters include bladder capacity (BC), pressure threshold (PT) at bladder capacity, bladder compliance (BC/PT), maximal voiding pressure, contraction duration, voided volume (VV), residual volume (RV), and voiding efficacy (VE = VV/(VV + RV)).

For the repeated CMG recordings, the CMG parameters were normalized to the measurement of the first control CMG. Repeated measurements in the same animal under the same experimental conditions were averaged. The results from different animals are reported as mean ± standard error (SE). Statistical significance (*P* < 0.05) was detected by Student *t*‐test or two‐way analysis of variance (ANOVA) followed by Bonferroni multiple comparison.

## Results

### TNS application during CMG did not alter micturition reflex

**Figure 1.**
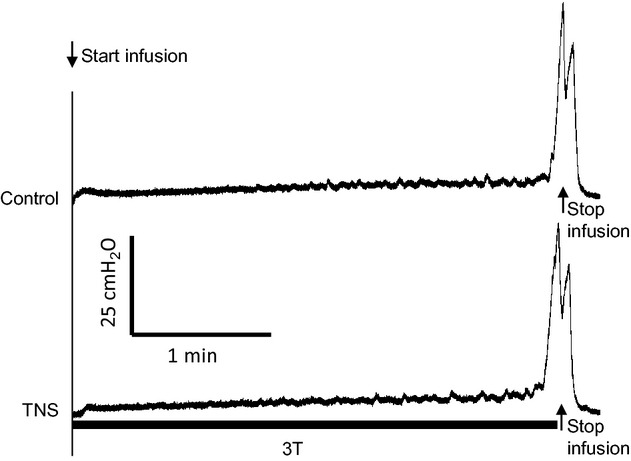
Representative CMGs with/without tibial nerve stimulation (TNS). The duration of stimulation is indicated by the black bar under the bladder pressure trace. T – threshold TNS intensity for inducing toe twitching. Stimulation: 5 Hz, 0.2 msec, *T* = 0.06 mA. Short arrows indicate the start and stop of the infusion. Infusion rate = 0.08 mL/min.

TNS at 5 Hz and 2–4T intensity (0.1–2.4 mA) had no effect on the micturition reflex when it was applied continuously during the CMG (Fig. 1). Table 1 summarizes the normalized results from 24 rats. There was no change in bladder capacity (control/TNS: 0.36 ± 0.04/0.37 ± 0.05 mL), voiding efficiency (80.9 ± 5.2/73.1 ± 7.8%), maximal voiding pressure (40.2 ± 1.6/40.8 ± 1.7 cmH_2_O), contraction duration (28.2 ± 2.5/27.7 ± 3.0 sec), and compliance (0.052 ± 0.005/0.048 ± 0.006 mL/cmH_2_O).

**Table 1.** Normalized CMG parameters obtained from the control CMGs without tibial nerve stimulation (TNS) or from the CMGs during TNS.

	Control CMG (mean ± SEM)	CMG during TNS (mean ± SEM)	*P*‐value
Bladder capacity (%)	97.1 **±** 2.59	97.7 **±** 7.46	0.9318
Voiding efficiency (%)	99.0 **±** 3.99	85.4 **±** 6.88	0.0966
Maximum voiding pressure (%)	98.1 **±** 1.09	99.9 **±** 2.20	0.3779
Contraction duration (%)	100.6 **±** 3.89	101.3 **±** 7.85	0.9051
Compliance (%)	96.6 **±** 2.99	90.5 **±** 7.65	0.3639

*N* = 24 rats for both control and TNS CMGs.

### Poststimulation inhibition induced by 30‐min TNS

**Figure 2.**
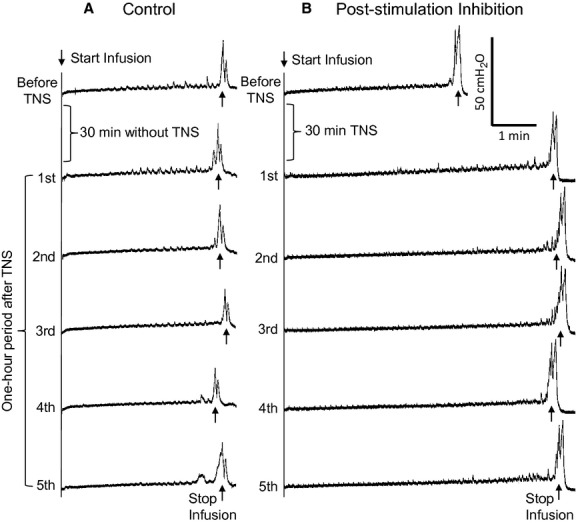
Poststimulation inhibition induced by 30‐min tibial nerve stimulation (TNS). (A) Repeated CMGs without 30‐min TNS. (B) Repeated CMGs before and after 30‐min TNS. TNS: 5 Hz, 0.2 msec, 4T = 0.28 mA. Infusion rate = 0.08 mL/min.

**Figure 3.**
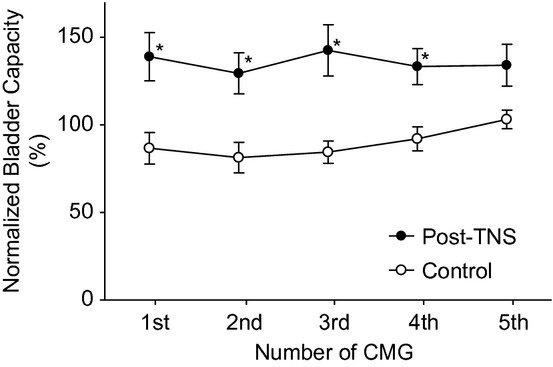
Poststimulation inhibition induced by 30‐min tibial nerve stimulation (TNS). Bladder capacity was significantly increased during the four repeated CMGs following the 30‐min TNS when compared to the control group without TNS. *Significantly different from control group (two‐way ANOVA with post hoc Bonferroni test). *N* = 12 rats for both control and post‐TNS groups. TNS: 5 Hz, 0.2 msec, 2–4T = 0.1–2.4 mA.

**Figure 4.**
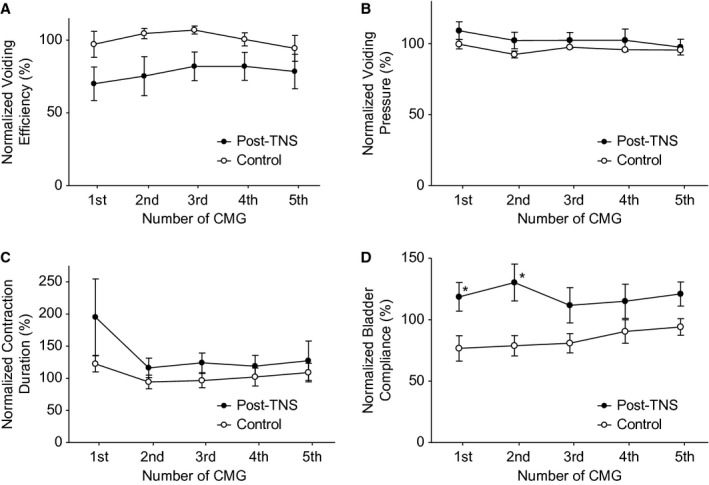
Poststimulation effect of 30‐min TNS on voiding efficiency (A), maximal voiding pressure (B), bladder contraction duration (C), and bladder compliance (D). *Significantly different from control group (two‐way ANOVA with post hoc Bonferroni test). *N* = 12 rats for both control and post‐TNS groups. TNS: 5 Hz, 0.2 msec, 2–4T = 0.1–2.4 mA.

Application of 30‐min TNS (2–4T) with the bladder empty induced poststimulation inhibition in the following four repeated CMGs performed during a period of approximately 50 min after the TNS was terminated (Fig. 2). Bladder capacity was significantly (*P* < 0.05) increased to about 140% of prestimulation level in the first four control CMGs (Fig. 3) and bladder compliance was also significantly (*P* < 0.05) increased in the first two control CMGs (Fig. 4D). Maximal voiding pressure, contraction duration, and voiding efficiency were not changed significantly after the 30‐min TNS (Fig. 4A–C), although a nonsignificant reduction (about 20–30%) in voiding efficiency did occur (Fig. 4A).

## Discussion

This study in rats showed that TNS failed to inhibit the micturition reflex when it was applied acutely during a CMG (Fig. 1 and Table 1). However, a 30‐min TNS applied with the bladder empty induced a long‐lasting post‐TNS inhibition and significantly increased bladder capacity and compliance (Figs. 2–4). These results indicate possible different mechanisms of action for acute‐ and post‐TNS inhibition, providing valuable information for understanding the clinical application of tibial neuromodulation in the treatment of OAB.

Previous studies in cats (Tai et al. 2011a, 2012) and human (Amarenco et al. 2003; Vandoninck et al. 2003) have shown that TNS applied during a CMG inhibited the bladder and significantly increased bladder capacity. A recent study (Su et al. 2012) in rats has also shown that TNS successfully inhibited bladder reflex contractions under isovolumetric conditions, but stimulation during a slow infusion CMG was not performed. Therefore, in this study it was unexpected that TNS applied continuously during a CMG could not inhibit the bladder and increase bladder capacity (Fig. 1 and Table 1). This unexpected result indicates that TNS might activate different mechanisms in rats than in cats and humans. However, there are also similarities for TNS in these species. In rats 30‐min TNS induced a sustained poststimulation inhibition that lasted for about 50 min (Figs. 2, 3). Similar poststimulation inhibition was also induced in cats by 30‐min TNS (Tai et al. 2011b). In humans, 30‐min TNS once a month produces a significant improvement of OAB symptoms (Peters et al. 2009, 2013). These results suggest that similar mechanisms of action for post‐TNS inhibition might exist in different species, but the mechanisms of action for acute‐TNS inhibition could be different.

It is difficult to understand how TNS applied continuously during a CMG does not inhibit the bladder but at the same time that it can induce a 50‐min long poststimulation inhibition after the termination of a 30‐min TNS. One possible explanation for this difference is that TNS inhibition gradually develops as the stimulation is continued during the 30‐min period. Recent studies in rats showed that TNS at 10 Hz quickly inhibited isovolumetric rhythmic bladder contractions within seconds after the start of the stimulation (Su et al. 2012). However, this rapid onset TNS inhibition gradually disappeared in about 5 min during continuous 15‐min stimulation (Su et al. 2013a). In our study the duration of each CMG was about 4–5 min. Therefore, it is possible that the TNS inhibition was lost during the slow infusion CMG before the bladder volume reached the threshold for triggering a micturition reflex. However, a longer stimulation duration (30 min) induced post‐TNS inhibition (Figs. 2, 3), suggesting that TNS inhibition reappears after a certain period of time. A recent report in rats (Su et al. 2013a) also showed that the TNS inhibition that disappeared during stimulation could reappear in about 10 min after termination of a 5‐min stimulation. These results suggest that further studies are needed to determine the time course of the development of TNS inhibition.

It is worth mentioning that in the previous rat study (Su et al. 2013a) a single‐cathodal electrode was placed on the tibial nerve with the anodal needle electrode inserted at the base of the tail. This electrode configuration could cause electrical current passing through the genital area, potentially stimulating the pudendal nerve branches. It is known that pudendal nerve stimulation can inhibit bladder activity. In our study, a tripolar cuff electrode was placed on the tibial nerve, which localized the stimulation current at the tibial nerve. In addition, the previous rat study (Su et al. 2013a) closed the urethral outlet in order to record isovolumetric bladder contractions, while in our study the urethral outlet was open preventing additional tests of TNS on isovolumetric contractions. Therefore, in future rat experiments aimed at determining the time course of acute‐TNS inhibition both electrode configurations should be used in order to fully understand the differences between the two sets of experiments.

It is also possible that the post‐TNS inhibition has a different mechanism other than the acute‐TNS inhibition. As TNS continues, the post‐TNS inhibition might gradually build up while the acute‐TNS inhibition is gradually lost. A previous study using rat spinal cord has shown that low‐frequency (0.2–1 Hz) stimulation of large afferent A‐fibers in the dorsal root reduced afferent‐induced excitatory postsynaptic potentials by 52% (Ikeda et al. 1999). Interestingly, this inhibition required 30 min to reach its maximum and lasted for more than 1 h. Additional studies to determine the potentially different time courses for acute‐ and post‐TNS inhibition are warranted. The possible different mechanisms underlying acute‐ and post‐TNS inhibition are important for understanding the therapeutic effect of clinical tibial neuromodulation of bladder overactivity.

Our recent studies in cats (Tai et al. 2012; Zhang et al. 2012; Matsuta et al. 2013) have shown that opioid receptors play an essential role in TNS inhibition of bladder overactivity and that activation of metabotropic glutamate subtype 2 or 3 receptors can modulate this TNS opioid inhibitory mechanism. It still needs to be determined whether these neurotransmitter receptors are also involved in TNS inhibition in rats. However, previous studies in rats have shown that endogenous opioids are released in the central nervous system (CNS) during electrical stimulation of somatic afferent nerves (Yaksh and Elde 1981; Han et al. 1991; Wang et al. 2005). A recent study in rats has also shown that *μ*‐opioid receptors are involved in sacral neuromodulation of bladder activity (Su et al. 2013b). In addition, whether these neurotransmitters play different roles in acute‐ and post‐TNS inhibition also needs to be determined. The animal models such as the one in this study will be very useful for examining the neurotransmitter mechanisms of tibial neuromodulation due to the potential toxicity of many drugs/chemicals for human testing.

The post‐TNS inhibition probably suppressed the afferent pathway of the micturition reflex rather than the efferent pathway, because maximal voiding pressure and bladder contraction duration were not changed once the micturition reflex was triggered (Fig. 4B and C). The voiding efficiency was also not changed significantly (Fig. 4A), indicating that TNS did not significantly influence the function of urethral sphincter. However, TNS significantly increased bladder compliance in the first two CMGs during the post‐TNS inhibition (Fig. 4D), indicating that the detrusor was more relaxed. Thus, it is possible that TNS might activate a reflex output to the bladder through the sympathetic nerves (hypogastric nerves) and relax the detrusor via *β* adrenergic mechanisms (Lindström et al. 1983). Additional electrophysiological studies are needed to investigate the neural pathways involved in TNS inhibition of micturition reflex.

A comparison of this study with a previous study by Su et al. (2013a) in rats reveals that TNS elicits two distinct types of bladder inhibition that are activated by different stimulation paradigms and have different onset and duration of action. These results suggest that different mechanisms might contribute to acute‐ and post‐TNS inhibition of reflex bladder activity. This study also reveals similarities and differences in TNS inhibition between different species and establishes a rat model for studying tibial neuromodulation. Understanding the mechanisms underlying acute‐ and post‐TNS inhibition is important for further improving the efficacy of tibial neuromodulation therapy or developing new treatments for OAB.

## Conflict of Interest

None declared.
